# Clinical features and imaging characteristics in achiasmia

**DOI:** 10.1093/braincomms/fcad219

**Published:** 2023-08-22

**Authors:** Anastasia Pilat, Rebecca J McLean, Anna Vanina, Robert A Dineen, Irene Gottlob

**Affiliations:** Department of Neuroscience, Psychology and Behaviour, University of Leicester, Leicester, UK; Department of Neuroscience, Psychology and Behaviour, University of Leicester, Leicester, UK; Eleanor Cross Healthcare, Northampton, UK; Division of Clinical Neuroscience, Queen’s Medical Centre, Radiological Sciences, University of Nottingham, Nottingham, UK; NIHR Nottingham Biomedical Research Centre, Queen’s Medical Centre, University of Nottingham, Nottingham, UK; Department of Neuroscience, Psychology and Behaviour, University of Leicester, Leicester, UK; Department of Neurology, Cooper University Hospital, Cooper Neurological Institute, Camden, USA

**Keywords:** OCT, MRI, achiasmia

## Abstract

Achiasmia is a rare visual pathway maldevelopment with reduced decussation of the axons in the optic chiasm. Our aim was to investigate clinical characteristics, macular, optic nerve and brain morphology in achiasmia. A prospective, cross-sectional, observational study of 12 participants with achiasmia [8 males and 4 females; 29.6 ± 18.4 years (mean ± standard deviation)] and 24 gender-, age-, ethnicity- and refraction-matched healthy controls was done. Full ophthalmology assessment, eye movement recording, a high-resolution spectral-domain optical coherence tomography of the macular and optic disc, five-channel visual-evoked responses, eye movement recordings and MRI scans of the brain and orbits were acquired. Achiasmia was confirmed in all 12 clinical participants by visual-evoked responses. Visual acuity in this group was 0.63 ± 0.19 and 0.53 ± 0.19 for the right and left eyes, respectively; most participants had mild refractive errors. All participants with achiasmia had see-saw nystagmus and no measurable stereo vision. Strabismus and abnormal head position were noted in 58% of participants. Optical coherence tomography showed optic nerve hypoplasia with associated foveal hypoplasia in four participants. In the remaining achiasmia participants, macular changes with significantly thinner paracentral inner segment (*P* = 0.002), wider pit (*P* = 0.04) and visual flattening of the ellipsoid line were found. MRI demonstrated chiasmatic aplasia in 3/12 (25%), chiasmatic hypoplasia in 7/12 (58%) and a subjectively normal chiasm in 2/12 (17%). Septo-optic dysplasia and severe bilateral optic nerve hypoplasia were found in three patients with chiasmic aplasia/hypoplasia on MRI. In this largest series of achiasmia patients to date, we found for the first time that neuronal abnormalities occur already at the retinal level. Foveal changes, optic nerve hypoplasia and the midline brain anomaly suggest that these abnormalities could be part of the same spectrum, with different manifestations of events during foetal development occurring with varying severity.

## Teaser

In this publication, Pilat *et al*. found changes at the retinal level in participants with achiasmia. A combination of the retinal changes, optic nerve hypoplasia and septo-optic dysplasia, was present in 33% of patients, suggesting possible different manifestations of disrupted events during foetal development occurring with varying severity.

## Graphical Abstract

**Graphical Abstract:**
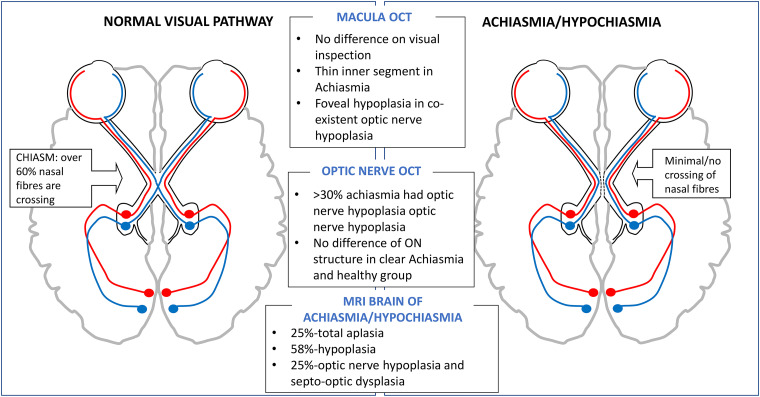

## Introduction

Typically, in the optic chiasm, ∼60% of ganglion cell axons from the nasal retinae cross to the contralateral brain, whereas the temporal axons remain uncrossed.^1^

Achiasmia is a rare visual pathway maldevelopment with reduced decussation of the axons in the optic chiasm.^2^ The literature to date, generally describes single patients with achiasmia,^3-8^ with a maximum of nine patients examined.^9^

The clinical characteristics of achiasmia include decreased vision, infantile see-saw nystagmus, strabismus and reduced or absent stereo vision.^2,10-12^ The mechanism of see-saw nystagmus remains unknown; however, it is related to conditions affecting the chiasm,^13,14^ possibly associated with vestibuloocular reflex impairment.^13,15^

Visual-evoked responses (VERs) are typically asymmetrical in achiasmia with larger amplitudes on the ipsilateral side of the stimulated eye and are diagnostic.^10,12,16-18^ Ipsilateral projection of visual stimuli has been confirmed with functional MRI studies.^12,16^

Despite severe abnormal anatomy of the visual pathway, relatively good visual functions and visual fields have been reported in achiasmia.^10,12,19^ Sami *et al*.^9^ described the imaging patterns of nine patients with chiasmatic hypoplasia as being associated with (i) normal optic nerve appearance (three out of nine patients); (ii) septo-optic dysplasia (SOD; two patients); (iii) other brain anomalies such as skull base encephalocele with agenesis of the corpus callosum (three patients).

MRI studies using diffusion-tensor imaging and tractography have demonstrated normal topography in the geniculo-striate projection and occipital callosal connections in achiasmia.^6,19^ Hoffmann *et al*.^19^ hypothesized that intracortical reorganization contributes to relatively normal visual function. Bao *et al*.,^4^ using functional MRI, showed the presence of closely located non-interacting neuronal populations sharing the same local vascular supply, resulting in an overlapped representation of the visual hemifields of both eyes.

Optical coherence tomography (OCT) allows an investigation of retinal and optic nerve structures at an almost microscopic level.^20,21^ Weiss *et al*.^8^ examined anatomical features of the macula in one patient with achiasmia using OCT and did not report any significant pathology.

To our knowledge, macular and optic nerve analysis has not been performed using a larger sample size in patients with achiasmia.

In the present study, the largest series of participants with achiasmia to date, we aim to investigate: (i) clinical characteristics [visual acuity (VA), refraction and strabismus/stereopsis]; (ii) chiasmatic appearances and associated visual pathway or brain abnormalities using MRI and (iii) description of macula and optic nerve structure using OCT compared with data from a healthy control group.

## Materials and methods

The study was approved by the local research ethics committee (East Midlands Ethic Committee, UK—Nottingham 2 REC REF 12/EM/0261, IRAS REF 105137) and adhered to the tenets of the Declaration of Helsinki. Informed consent was obtained from all participants, or for children, from their parents/carers. The data are not publicly available due to information that may compromise the privacy of participants.

### Participants

All participants with achiasmia were recruited from the Neuro-Ophthalmology clinic at the Leicester Royal Infirmary, UK and had a standard ophthalmologic examination, including refraction, best-corrected VA [measured with a logMAR test using computerized software package Precision Vision Visual Acuity Testing (Precision Vision, La Salle, IL, USA)], refraction, orthoptic examination, slit-lamp examination, intraocular pressure measurements and dilated fundoscopy. Stereopsis testing was performed using Frisby test (Stereotest Ltd, UK) for adults and Lang test for children (Lang-Stereotest, Switzerland). Two-dimensional (horizontal and vertical) eye movements were recorded in all participants with achiasmia (EyeLink 2, SR Research Ltd, Osgoode, Canada and EyeLink 1000, SR Research Ltd, Osgoode, Canada for Participant 12) under binocular conditions and with either eye occluded.

Five-channel VER testing was performed (Medelec Synergy; Oxford Instruments, Oxfordshire, UK) with standard International Society for Clinical Electrophysiology of Vision protocol for diagnostic purposes.^22^

The diagnosis of achiasmia was based on the presence of see-saw nystagmus and reduced/absent decussation in the chiasm on VER.^9^

In addition, 24 gender-, ethnicity-, age- and refraction-matched healthy controls, who had no known eye pathology, no previous intraocular surgery, no neurological pathology, diabetes or active cardiovascular disease, were recruited as controls for the OCT data.

### Optical coherence tomography

High-resolution spectral-domain OCT (Copernicus; Optopol Technology S.A., Zawiercie, Poland; wavelength = 850 nm; theoretical axial resolution of 3.0 μm), to acquire tomograms (7 × 7 × 2 mm, 75 B-scans, 743 A-scans per B-scan), centred on the fovea and optic nerve, was performed on adult participants. In two participants (Participants 11 and 12), SD-OCT images were obtained using a hand-held device (HH-OCT, Envisu 2300, Leica Microsystems) with a wavelength of 840 nm, theoretical axial resolution of 2.4 μm (10 × 10 × 2.46 mm, 100 B-scans and 500 A-scans per B-scan).^23^ The consistency of measurements between the two instruments was demonstrated by high interclass correlations in a previous publication.^24^

An analysis of single tomograms of the fovea and optic nerve was masked, with random numbers allocated to scans of participants and control subjects, and was performed by the same examiner (A.P.).

### Foveal analysis

Foveal analysis was performed using a single central horizontal flattened B-scan at the deepest point of the foveal pit, where the outer segments (OSs) of photoreceptors were thickest. Analysis was conducted using an ImageJ macro (http://imagej.nih.gov/ij/; provided for use in the public domain by the National Institute of Health, Bethesda, MD, USA, downloaded April 2012).

**Figure 1:**
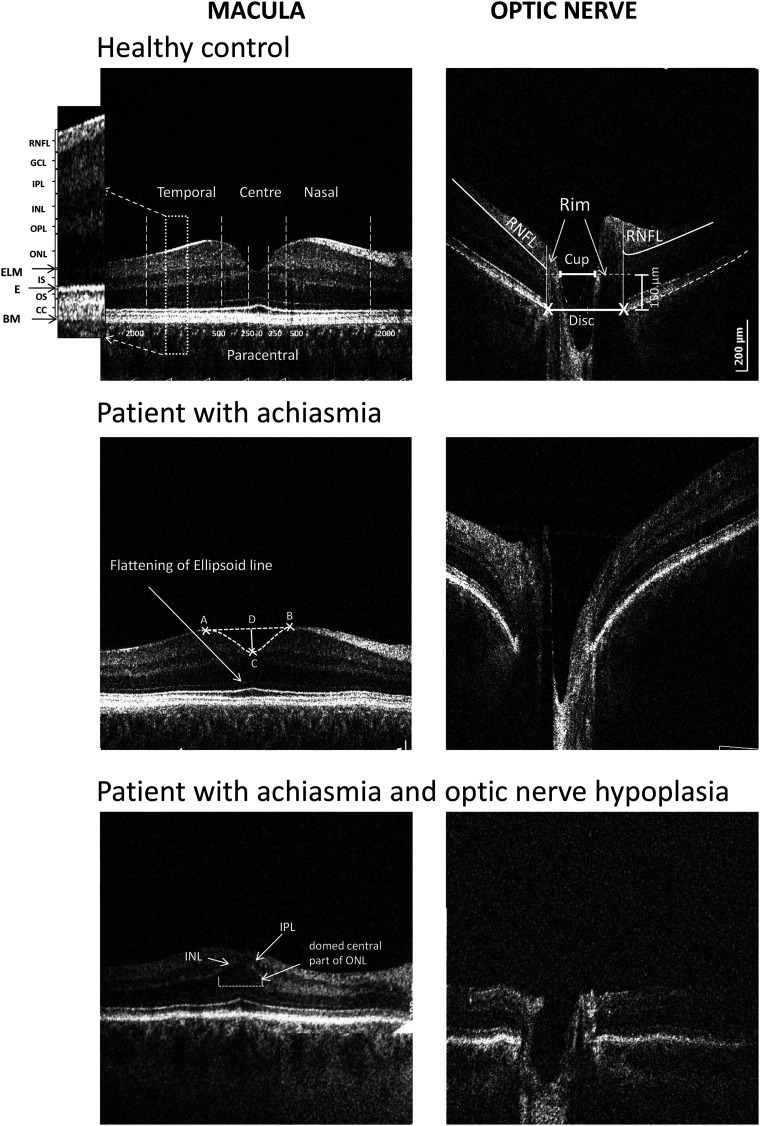
**Horizontal spectral-domain optical coherence tomography B-scan images of the macula area and the optic nerve head of a control subject (A and B, respectively), a participant with achiasmia (C and D) and a participant with achiasmia and co-existent ONH (E and F).** (**A**) The position of the different retinal layers: RNFL, retinal nerve fibre layer; GCL, ganglion cell layer; IPL, inner plexiform layer; INL, inner nuclear layer; OPL, outer plexiform layer; ONL, outer nuclear layer; ELM, external limiting membrane; IS, inner segment; E, ellipsoid; OS, outer segment; BM, Bruch’s membrane. The thickness of the layers was measured in the centre of the fovea, in the paracentral area (from 250 µm nasally to 250 µm temporally) and nasally and temporally (from 500 to 2000 µm). The horizontal macula scan of the participant with achiasmia (**C**) showed a flattening of the ellipsoid line. The participant with co-existing ONH (**E**) has features typical of macula hypoplasia (thin retina, flat foveal pit, thin RNFL and GCL, continuation of the IPL and INL, domed central ONL and absence of the upward deflection of the ellipsoid line). The foveal pit parameters: AB, foveal width, i.e. the lateral distance measured between the most prominent nasal and temporal points on the ILM; DC, pit depth, corresponding to the axial distance from the line connecting the most prominent nasal and temporal ILM points to the bottom of the pit; triangle ABC = pit area, limited by ILM and pit width. Optic disc/cup size, peripapillary RNFL thickness of the participant with both achiasmia and ONH (**F**) are considerably reduced.

The borders of the following retinal layers were used to calculate thickness measurements: retinal nerve fibre (RNFL), ganglion cell layer (GCL), inner plexiform layer (IPL), inner nuclear layer (INL), outer plexiform layer (OPL), outer nuclear layer (ONL), inner segment (IS), OS, interdigitation zone and retinal pigment epithelium (RPE) layers, using the protocol from one of our previous studies.^23^ Retinal layer thickness was measured across the whole scan [central point, paracentral (averaged thickness of each layers from 250 µm nasally to 250 µm temporarily from the centre) and nasal/temporal areas (averaged thickness of each layers from 500 to 2000 µm from the centre, nasally and temporarily); Fig. 1A].

For foveal pit parameters, the lateral distance between the largest nasal and temporal retinal thickness points of the internal limiting membrane (ILM, foveal width); axial distance from the line connecting the largest nasal and temporal retinal thickness points to the bottom of the pit (pit depth) and pit area (area, limited by ILM and pit width) were measured (Fig. 1C).

### Optic nerve head analysis

Optic nerve analysis was performed using an ImageJ macro (National Institute of Health; available at: http://rsbweb.nih.gov/ij/; accessed 3 March 2014) on a flattened B-scan through the deepest point of the optic nerve cup. Disc diameter margins were identified as the edges of the retinal pigment epithelium manually. The cup diameter (offset 150 µm anteriorly to the disc axis), cup depth and horizontal rim size (distance between horizontal disc and cup diameters) and temporal and nasal height (between the horizontal cup diameter level and RNFL, limited on the periphery by the disc margins) were measured automatically by the macro. Peripapillary RNFL thickness was measured in a region from 1200 to 1600 µm on both sides of the centre of the cup (same as the default setting in the Optopol automated analysis).

### Magnetic resonance imaging

Participants with achiasmia, who were able to attend the Leicester Royal Infirmary for the MRI scan, underwent scanning at 1.5 T (Siemens Symphony, Erlangen, Germany), including sagittal MPRAGE (TR/TE/TI 1850/3.9/1100, flip angle = 15°, base resolution = 256, FOV = 300, voxel size 1.2 mm × 1.2 mm × 1.2 mm) and axial constructive interference in the steady state (CISS; Repetition Time/Time to Echo 11.4/5.7, flip angle 70°, base resolution = 256, Field-of-view = 200, voxel size 0.8 × 0.8 × 1 mm). Two participants were not able to attend Leicester Royal Infirmary for MRI; therefore, their previous clinical MRI scans were accessed and reviewed.

For clear chiasm visualization, scans were reformatted using Voxar software (Barco, Kortrijk, Belgium) into true sagittal images, angled axial and coronal images. Images were reviewed and classified by an experienced accredited neuroradiologist (R.A.D.) as showing chiasmatic aplasia (complete absence of the chiasm), chiasmatic hypoplasia (chiasm present but of small size) or subjectively normal chiasm. Other imaging findings, such as optic nerve hypoplasia (ONH), pituitary abnormalities or other brain malformations were also recorded.

### Statistical analysis

Statistical analysis was performed using SPSS software version 16.0 (SPSS Inc., Chicago, IL, USA). Optic nerve head and macular data were normally distributed (Shapiro–Wilk test). The parameters of ONH and macula were analysed using a linear mixed model. Bonferroni correction was applied for multiple comparisons. *T*-test was used to compare macula pit parameters. A two-tailed paired *t*-test was used to assess any asymmetry of the layer thickness between the nasal/temporal macular areas. Spearman’s correlation coefficient was used for correlation analysis. *P* ≤ 0.05 was considered statistically significant.

## Results

### Clinical characteristics of participants with achiasmia

Twelve participants with achiasmia (8 males and 4 females; mean age = 29.6 years; SD ± 18.4 years) and 24 healthy controls (16 males and 8 females; mean age = 26.5 years; SD ± 14.6 years) were included in the analysis. Clinical-demographic data of the achaismic participants are provided in Table 1.

**Table 1:** Age, gender, ethnicity and clinical data of the participants with achiasmia

Participant’s number	Age (years)	Gender	Ethnicity	LogMar visual acuity		Refraction (spherical equivalent)		Ophthalmic pathology	Nystagmus/head posture (−no head posture)	MRI findings
				RE	LE	RE	LE			
1	9	M	C	0.50	0.20	−0.50	−0.50	BE ONH	SS/left head tilt	Chiasmatic hypoplasia
2	21	M	A	0.30	0.50	−2.50	−2.25		SS/left head turn	Chiasmatic hypoplasia
3	23	M	C	0.80	0.80	+3.00	+3.00	RCS	SS/left head tilt	Chiasmatic aplasia, small lipoma related to pituitary stalk
4	29	M	C	1.00	0.60			BE ONH; RCS	SS/–	Chiasmatic aplasia
5	27	M	C	0.60	0.60			BE ONH; Exophoria	SS/–	Chiasmatic hypoplasia, bilateral ONH
6	44	F	C	0.80	0.80	−2.00	−2.00		SS/left head tilt	Chiasmatic hypoplasia
7	30	M	AC	0.60	0.60				SS/left head turn	Chiasmatic aplasia
8	61	M	C	0.60	0.60	−12.50	−11.00	BE ONH; LCS	SS/–	Chiasmatic hypoplasia, bilateral ONH, SOD, abnormal adhesions crossing anterior third ventricle, bilateral perisylvian polymicrogyria
9	44	F	C	0.80	0.20			RCS, R/L	SS/alternating head turn	Normal chiasm
10	55	M	C	0.50	0.50	+1.75	+1.50	LDS	SS/–	Chiasmatic hypoplasia
11	10	F	C	0.50	0.50	−3.25	−3.25	V pattern exotropia	SS/left head turn	Normal chiasm
12	2	F	AC	FF	FF	+2.00	+2.00	RCS	SS/–	Chiasmatic hypoplasia, bilateral ONH, small pituitary gland

A, Asian; AC, Afro-Caribbean; BE, both eyes; C, Caucasian; F, female; FF, fix and follow; LE, left eye; M, male; ONH, optic nerve hypoplasia; R or L C/D S, right or left convergent/divergent squint; RE, right eye; R/L, right hypertropia; SOD, septo-optic dysplasia; SS, see-saw nystagmus.

**Figure 2:**
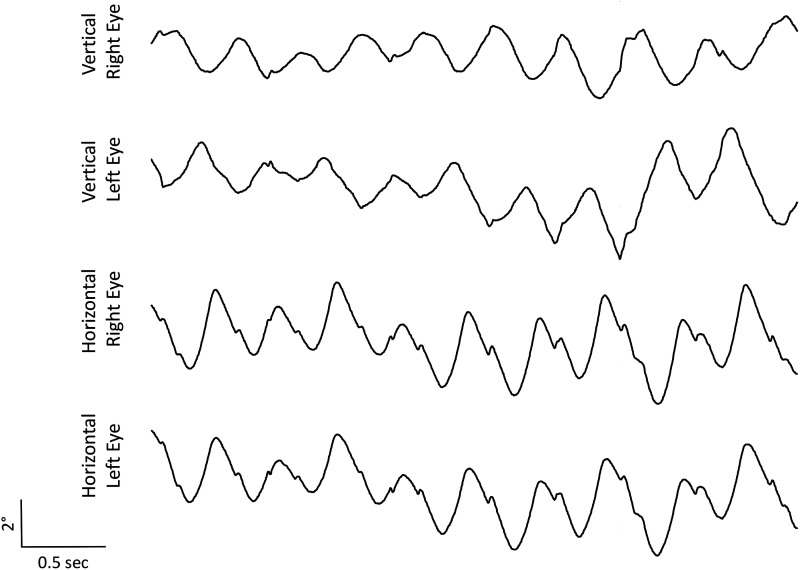
**Horizontal and vertical eye movement recordings from Participant 7, demonstrating see-saw nystagmus.** For horizontal eye traces, an upward movement denotes the eyes moving to the right and a downward movement denotes the eyes moving to the left; for vertical eye traces, an upward movement denotes the eyes moving upwards and a downward movement denotes the eyes moving downwards. Horizontal eye movements were conjugate, whereas vertical eye movements were dysconjugate. Clinically upward movements of the eyes were associated with intorsion and downward eye movements with extorsion (torsional eye movements are not shown in this figure).

None of the participants had any dysmorphic features, congenital anomalies or developmental delay. The presence of see-saw nystagmus on initial examination prompted further testing, which included VER to confirm the diagnosis of achiasmia. All 12 participants demonstrated see-saw nystagmus of a predominantly pendular waveform. Video 1 shows a typical see-saw nystagmus in achiasmia, a disconjugate eye movement. One eye moves upwards and intorts, and the other eye moves downwards and extorts (Participant 7). An example of eye movement recordings from Participant 7 is shown in Fig. 2.

In 7 of 11 participants (58%), nystagmus was associated with moderate-to-severe anomalous head posture, mostly with a turn or tilt to the left. Seven participants had apparent eye misalignments of varying degrees, although small degrees of strabismus cannot be excluded in the remaining participants due to the presence of see-saw nystagmus. Stereopsis testing was negative for all participants.

Clinical examination of the participants with achiasmia revealed the presence of bilateral ONH in four participants. This finding was confirmed by OCT (Fig. 1E and F) and MRI (Participant 8, Table 1).

The average logMAR VA in participants with achiasmia was 0.63 ± 0.19 and 0.53 ± 0.19 for the right and left eyes, respectively. Four participants with co-existent ONH had significantly lower VA (0.73 ± 0.23, *P* = 0.01 and 0.60 ± 0.10, *P* = 0.08 for right and left eyes, respectively) when compared with isolated achiasmia. Participants did not have VA asymmetry between the right and left eyes (*P* = 0.61).

Mild refractive abnormality recorded between −3.25 and +3.00 Dioptres (mean = −0.13 ± 1.92 and −0.13 ± 1.86 Dioptres for the right and left eyes, respectively) was found in 11 participants. Participant 8 was the exception and severely myopic (>10.00 Dioptres). No correlation was found between refractive error and VA.

### Visual-evoked responses

All participants with isolated achiasmia showed a larger positive peak on flash and pattern-onset/offset VER over the ipsilateral occiput. Changes were less obvious with a smaller amplitude difference in hypochiasmia (*r_s_* = 0.68, *P* < 0.02; Supplementary Fig. 1). All patients showed interocular inverse polarity. Participants with coexisting ONH had reduced amplitude when compared with the reference range. No correlation was found between VER parameters and OCT data in participants with achiasmia and no ONH.

### Optical coherence tomography

#### Foveal morphology

A visual inspection of the horizontal macular SD-OCT scans of eight patients (Fig. 1C) demonstrated minimal changes in the fovea. Participants with achiasmia had a flatter ellipsoid line in the paracentral area (Fig. 1C) when compared with healthy controls. In the four participants with ONH (Patients 1, 4, 5 and 9; Table 1), the parafoveal retina was thin, mainly due to thinning of RNFL, GCL with a flat pit. In the central fovea, four participants with ONH had foveal hypoplasia with continuation of GCL, IPL, INL, no updrift of the ellipsoid line and a ‘domed’ ONL (Fig. 1E and F). In all four participants with severe foveal changes, the ONH was diagnosed clinically and confirmed with VER testing. The foveal OCTs of the four participants with ONH were excluded from quantitative analysis, since the condition is associated with severe retinal changes (Fig. 1E).

**Figure 3:**
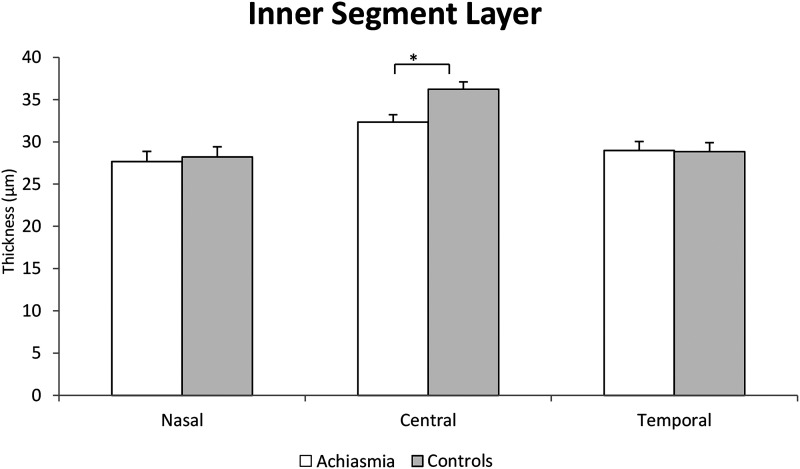
**Schematic diagram of the inner segment layer thickness (mixed model ANOVA) in participants with achiasmia and healthy controls (*f* = 12.89; *P* = 0.002).** Error bars represent standard deviations. *Significant difference (*P* ≤ 0.05).

Participants with achiasmia had a non-significant trend to a thicker central retina [240.14 ± 15.13 and 227.77 ± 21.03 µm in controls, *P* = 0.99 (linear mixed model)]. The difference was minimally significant with *t*-test (*P* = 0.04), corresponding to a smaller foveal pit (0.081 ± 0.025 versus 0.088 ± 0.017 mm^2^, *P* = 0.37 for pit area; 0.103 ± 0.025 versus 0.109 ± 0.019 mm, *P* = 0.21 for pit depth and 2.055 ± 0.188 versus 2.228 ± 0.194 mm, *P* = 0.04 for pit width). In the remaining eight participants, statistical analysis of retinal layer thickness revealed only significant thinning of the central IS in participants with achiasmia (*P* = 0.002, Fig. 3). No other retinal layers in the macula showed any statistically significant differences.

This thinning of IS was associated with flattening of the ellipsoid line (Fig. 1C and Supplementary Fig. 2). There was no correlation between the thickness of the IS layer and VA. The difference between thickness in the nasal/temporal of the GCL was statistically significant in the GCL of both groups (*P* < 0.001 and *P* = 0.008 for achiasmia and controls, respectively).

#### Optic nerve head morphology

Four patients had co-existing ONH with a smaller disc, cup diameters, cup depth and thin RNFL (Fig. 1F) and were subsequently excluded from statistical analysis. A visual inspection of ON scans of the remaining patients did not reveal any apparent differences between the achiasmia and the control groups and no statistical differences were found. OCT data for optic nerve parameters are shown in Supplementary Fig. 3.

Peripapillary RNFL thickness, measured temporally and nasally, did not reveal any statistical differences (*P* = 0.67 and *P* = 0.78, respectively).

#### Magnetic resonance imaging

**Figure 4:**
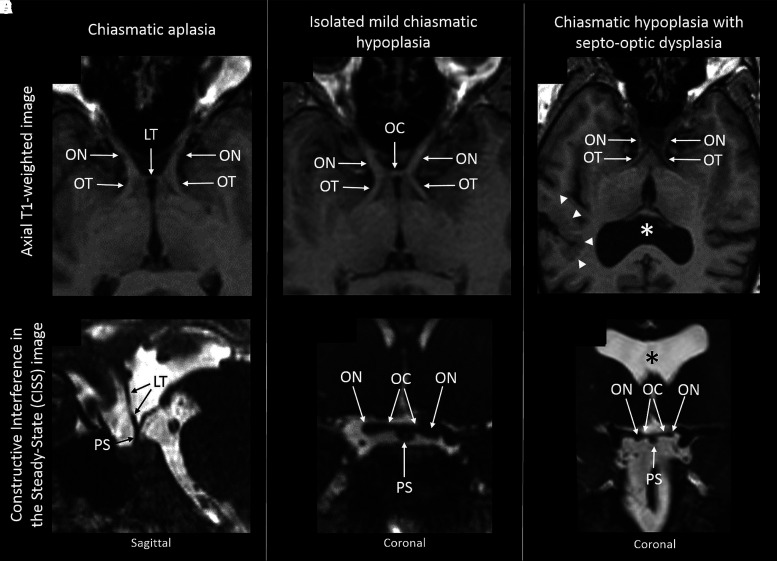
**MRI findings in achiasmic patients.** (**A**) Axial T_1_-weighted and (**D**) sagittal CISS images for a patient with chiasmatic aplasia (Patient 7). Note the widely separated optic nerves (ONs) and tracts (OTs), and complete absence of the optic chiasm (OC) with the lamina terminalis (LT) continuing vertically downwards to the pituitary stalk (PS). (**B**) Axial T_1_-weighted and (**E**) coronal CISS for a patient with chiasmatic hypoplasia (Patient 2). Note the normal volume of the OTs and ONs but the OC is wide and slender. (**C** and **F**) Comparable images from a patient with chiasmatic hypoplasia in the context of SOD (Patient 8). Note the marked hypoplasia of the OTs, ONs and OC, the absent septum pellucidum (asterisk) and perisylvian polymicrogyria (present bilaterally but only on the right side shown, white arrowheads).

Three participants (25%) had chiasmatic aplasia (i.e. no identifiable chiasm). Axial scans showed separated optic nerves in the suprasellar cistern, and a straight, vertical configuration of the lamina terminalis continuous with the pituitary stalk, with no intervening chiasm (Fig. 4A and D). One of these participants (Participant 3) was noted to have a small lipoma related to the pituitary stalk. Participant 4 had ONH which was apparent on clinical examination and OCT but subjectively normal optic nerve size on MRI.

In seven participants, the chiasm was present but variably hypoplastic (Fig. 4B and E). In two of these participants (Patients 5 and 12), a degree of ONH was also observed on MRI, clinical examination and OCT. Participant 12 also had a small pituitary gland. Participant 1 had ONH features on clinical and OCT examinations only. In Participant 8, chiasmatic hypoplasia and ONH were associated with SOD with the absence of the septum pellucidum (Fig. 4C and F) and co-existent bilateral perisylvian polymicrogyria.

Two participants had a subjectively normal chiasm size on MRI. We did not identify any significant correlations between the severity of structural chiasmatic abnormalities (aplasia or hypoplasia) and VA with retinal and optic nerve structure on OCT.

## Discussion

Achiasmia is a rare condition associated with infantile nystagmus (IN). Previously, retinal and optic nerve structures had not been systematically investigated.^9^ This prospective, cross-sectional, observational study describes the detailed ocular examination, including retinal and optic nerve OCT imaging, of the largest series of participants with achiasmia to date.

### Clinical characteristics (VA, refraction, strabismus/stereopsis)

In our study group of participants with achiasmia, we found a VA of 0.63 ± 0.19 and 0.53 ± 0.19 (logMAR) for the right and left eyes, respectively. Participants with co-existent ONH had slightly lower VA (0.73 ± 0.23 and 0.6 ± 0.01, for the right and left eyes, respectively). VA in IN is defined by multiple factors that could be affected by presence/absence of strabismus, amblyopia and severity/type of nystagmus and foveal and ONH.^23,25,26^ In albinism, one of the most common diseases associated with IN, VA is variable^27^ and reported to be primarily related to the severity of the foveal hypoplasia. VA in patients with IN and ONH has been reported to be mainly dependent on the structure of the fovea.^23,27-31^ However, our participants with achiasmia, and normal optic nerve size, had only minimal foveal changes despite demonstrating relatively low VA. Reduction of VA in achiasmia may therefore be related to multidirectional, including torsional, predominantly pendular nystagmus waveforms with poor foveation and/or by abnormal development of the visual pathway with intracortical reorganization.^19^

In our study, achiasmatic participants were mildly myopic. In contrast, with albinism, idiopathic infantile nystagmus (refraction varies in different publications from −0.65, −1.37 Diopters^28^ to moderate hyperopia^29^) and ONH who are mildly hyperopic. None of the participants in our study had detectable stereopsis. This is most likely caused by strabismus, disconjugate nystagmus, optic nerve fibres projecting to the ipsilateral side of the cortex or a combination of factors. Ipsilateral fibre projection integrated cortical information in achiasmatic patients would create a sensory conflict that is successfully prevented by a selective sensory block due to cortical plasticity.^2^

### Visual-evoked responses

VER in the achiasmic participants was comparable with descriptions in the literature, showing, in the main, a higher amplitude on the ipsilateral stimulation and intraocular inverse polarity.^11-13^ Patients with hypochiasmia had a less obvious difference in the positive peak between the eyes. This could be explained by the presence of some, albeit reduced crossing of fibres in the chiasm in hypochiasmia.

### Optical coherence tomography and MRI data

In 75% of achiasmic participants, the optic nerve head was normal on both clinical examination and OCT, even though the optic nerve pathway is severely altered at the level of the chiasm. Interestingly, three participants with achiasmia (25%) also had ONH confirmed with both OCT and MRI. One participant with clinical, OCT and VER confirmed ONH had a normal appearance of the nerve on MRI. All participants with ONH had a smaller disc, cup diameters, cup depth and thin RNFL. Additionally, in participants with ONH, foveal OCT showed features of severely altered ‘arrested’ foveal development, typical for patients with isolated ONH.^23^

Table 2 displays publications reporting achiasmia, and/or hypochiasmia, associated with other structural abnormalities. Despite the limited data, as in our study, the most common association of achiasmia was midline brain defects. Therefore, the presence of ONH and midline defects in a third of our patients suggests possible common mechanisms of developmental pathway alteration.

**Table 2:** Conditions/syndromes associated with achiasmia/hypochiasmia

Overlapping conditions/syndromes	Number of patients reported	Authors
Optic nerve hypoplasia and midline defects (absent septum pellucidum and hypopituitarism)		
Unilateral optic nerve aplasia	1	Handley *et al*.^30^
Optic nerve hypoplasia, absence of the septum pellucidum, thinning of the corpus callosum	1	Rudich and Lesser^31^
Combination of absent septum pellucidum, optic nerve hypoplasia and hypopituitarism	2	Sami *et al*.^9^
Microphthalmia with optic nerve hypoplasia	1	Giancipoli *et al*.^32^
Association with clefting disorders and encephaloceles		
Clefting disorders, encephalocele and agenesis of corpus callosum	3	Sami *et al*.^9^
Midline craniofacial cleft	1	Leitch *et al*.^33^
Other associations		
VACTERL syndrome: vertebral defects, anal atresia, cardiac defects, tracheoesophageal fistula, renal defects and limb defects	1	Prakash *et al*.^7^
Kapur–Toriello syndrome: cleft lip and palate that had undergone repair and rhinoplasty, anal atresia, vesicoureteral reflux, hypospadias, 11 ribs, growth hormone deficiency, hypothyroidism, pes planus and transsphenoidal encephalocele	1	Justin *et al*.^34^
Oesophageal atresia	1	Pensiero *et al*.^35^

Optic nerve development begins around the fourth week of gestation, in the substance of the optic stalk. Between the fourth and sixth weeks of foetal development, the chiasm structure starts to form by retinal ganglion cells axons.^36^ It is believed that non-crossing axons reach the chiasm earlier, whereas axonal decussation appears in a pulse manner, displaying long pauses in activity. The direction of the axons (crossed or uncrossed) is determined by multiple factors, for example Zic2 (expressed in retinal cells), spatial and temporal expression of the *Pax2* gene (expressed by growing axons), the *sonic Hh* gene (expressed by growing chiasm), express *Eph/ephrinA* molecules (expressed by glial cells) and metalloproteases. At the end of the third month of gestation, the chiasm reaches an adult-like hemidecussation.^37^

In 96% of normal foetuses, a developed chiasm can be measured at 20–31 weeks^38^ by ultrasound, and at 21 weeks by MRI.^39^ Therefore, the possible timeframe for altered chiasm formation is from the 4–6th to the 20–21st weeks of gestation.

The pathogenesis of ONH is still unknown. Failure of GCL differentiation or excessive RNFL regression during pregnancy are postulated to play an important role.^40,41^

As for foveal development, at 20–22 weeks of gestation only a single layer of cones is identified. From 25 weeks to 15–45 months after birth centripetal inner layer displacement (GCL, IPL and INL) with pit formation and IS/OS thickening taking place.^36^ In our study, participants with co-existent ONH have severely arrested foveal development that is also typical for isolated ONH (RNFL, GCL thinning, continuation of the GCL, IPL and OPL with ‘domed ONL’ in the central fovea).^23^

In contrast, patients with no ONH had only thinning and ‘flattening’ of IS in the fovea with an almost normal macular structure. Previously, IS changes were reported in patients with amblyopia with reasonably preserved macula.^42^ However, in contrast with amblyopia, no foveal pit changes were found in isolated achiasmia. Only pit width and central retinal thickness were significantly smaller in achiasmia patients when compared with controls (*P* = 0.04 and *P* = 0.04, respectively). It is possible, that pit formation was influenced by amblyopia. However, pit change could also be attributed to arrested foveal development, present to a lesser degree in achiasmia than in patients with ONH.

Macular findings in achiasmia also differ from the foveal hypoplasia changes in albinism, another condition associated with chiasmal misrouting whereby, in contrast to achiasmia, more fibres cross to the opposite hemisphere. Patients with albinism also have severe distinctive changes in macula with continuation of the inner retinal layers in the foveal pit with widening of the outer nuclear layer and OS.^23,43^ Recent studies also describe a possible nasal/temporal asymmetry of the GCL thickness of the fovea in albinism.^44,45^ We also found a nasal/temporal GCA difference in achiasmia. However, a similar GCA difference was also noted by us, as well as by other groups, in a healthy population.^46^ It is possible that this asymmetry would be more noticeable in achiasmia with volumetric analysis; however, this is currently unavailable due to acquisition speed of the available devices and the presence of see-saw nystagmus in this cohort. Therefore, the most striking difference in macular morphology with continuation of the inner retinal layers in albinism and almost normal macular structure in achiasmia suggests a different underlying mechanism^23^ rather than a similar one.

A co-existence of changes at varying levels of the optic pathway, including the fovea, optic nerve and brain, suggests a common mechanism in ONH and achiasmia. This common mechanism may act during pregnancy and interfere with normal development and therefore may be part of the spectrum of alteration. Severity, as well as duration and timing of a putative triggering factor, inducing alteration, could determine which brain and ocular structures are affected.

Our study represents the largest cohort of patients with achiasmia that have been imaged using OCT. However, achiasmia may not be adequately represented by the present sample size (*n* = 12). A further limitation of the study was that OCT analysis was based on single horizontal B-scan images rather than volumetric analysis. Single B-scan images were used due to the presence of see-saw nystagmus, with a torsional component, making realignment of B-scans extremely difficult. Consequently, described changes may not represent all the structural abnormalities in patients with achiasmia that could be captured by volumetric analysis. Therefore, with improvement in the acquisition speed of OCT images, volumetric analysis could provide additional information in future studies.

To date, all achiasmia cases previously described were considered sporadic.^7,30,33-35^ The literature about ONH is more controversial with multiple aetiologies being reported, including mutations in *PAX6*, *HESX1* and *SOX2* genes or various environmental factors such as maternal diabetes and foetal alcohol syndrome.^47^ Further genetic and foetal development studies are needed to further understand these conditions that could be part of the same spectrum.

## Conclusion

We have demonstrated, for the first time, changes at a retinal level in achiasma. Co-existing foveal changes, ONH and SOD in our series were present in 33% of patients. Together with the cases described in the literature, our data suggest that these abnormalities could be part of a spectrum with different manifestations of events during foetal developmental occurring with varying severity.

## Supplementary Material

fcad219_Supplementary_Data

## Data Availability

The data that support the findings of this study are available from the corresponding author, upon request.
