# Supplementary material for: Clinical features and imaging characteristics in achiasmia
Source: Brain Commun. 2023 Aug 22;5(4):fcad219. doi: 10.1093/braincomms/fcad219 (PMC10481774; doi:10.1093/braincomms/fcad219)
Supplement: fcad219_Supplementary_Data [file fcad219_Supplementary_Data.pdf]

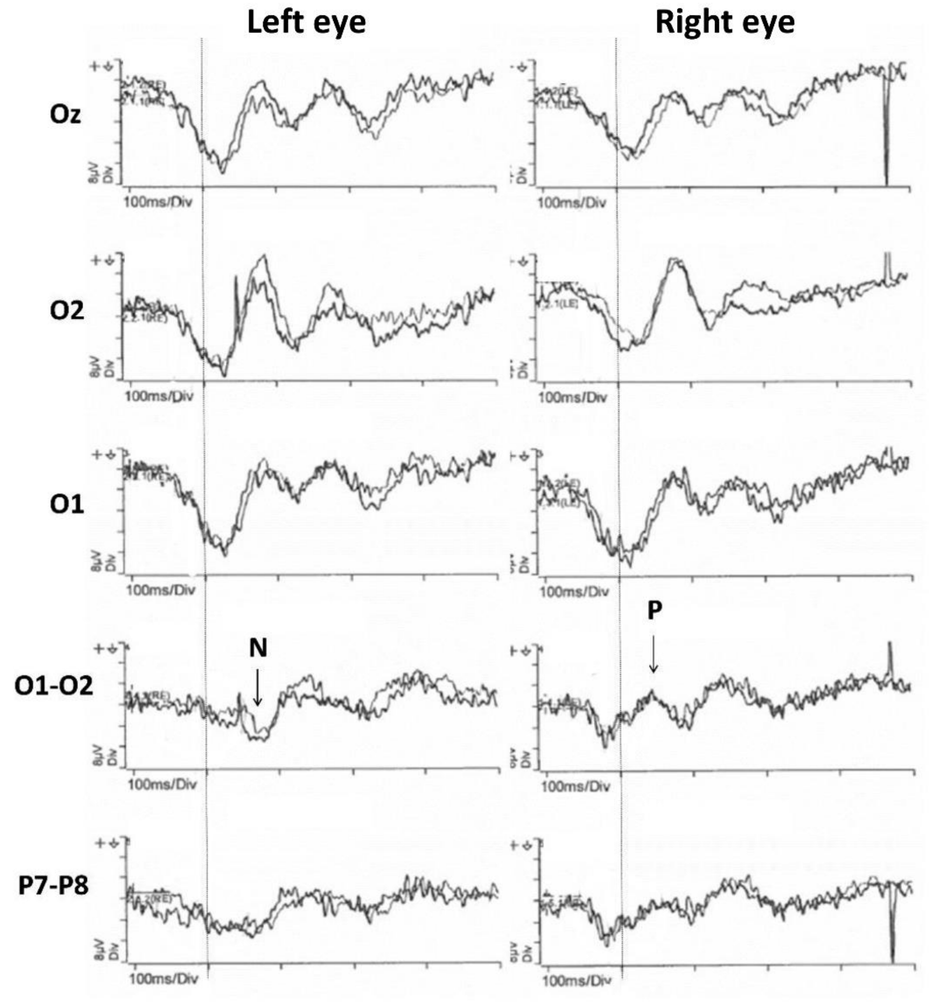

Supplementary figure 1: Visual Evoked Responses recording in an hypochiasmia (participant 11) of monocular left and right eye full-field pattern-onset stimulation. Oz= active/positive electrode, O1 and O2 = lateral electrodes, PO7 and P08 =additional lateral electrodes. N=negative wave, P=positive wave. Recordings for each electrode placed across the occiput are displayed from left hemisphere (O1, P7) to right hemisphere (O2 and P8). Arrows show interocular inverse polarity.

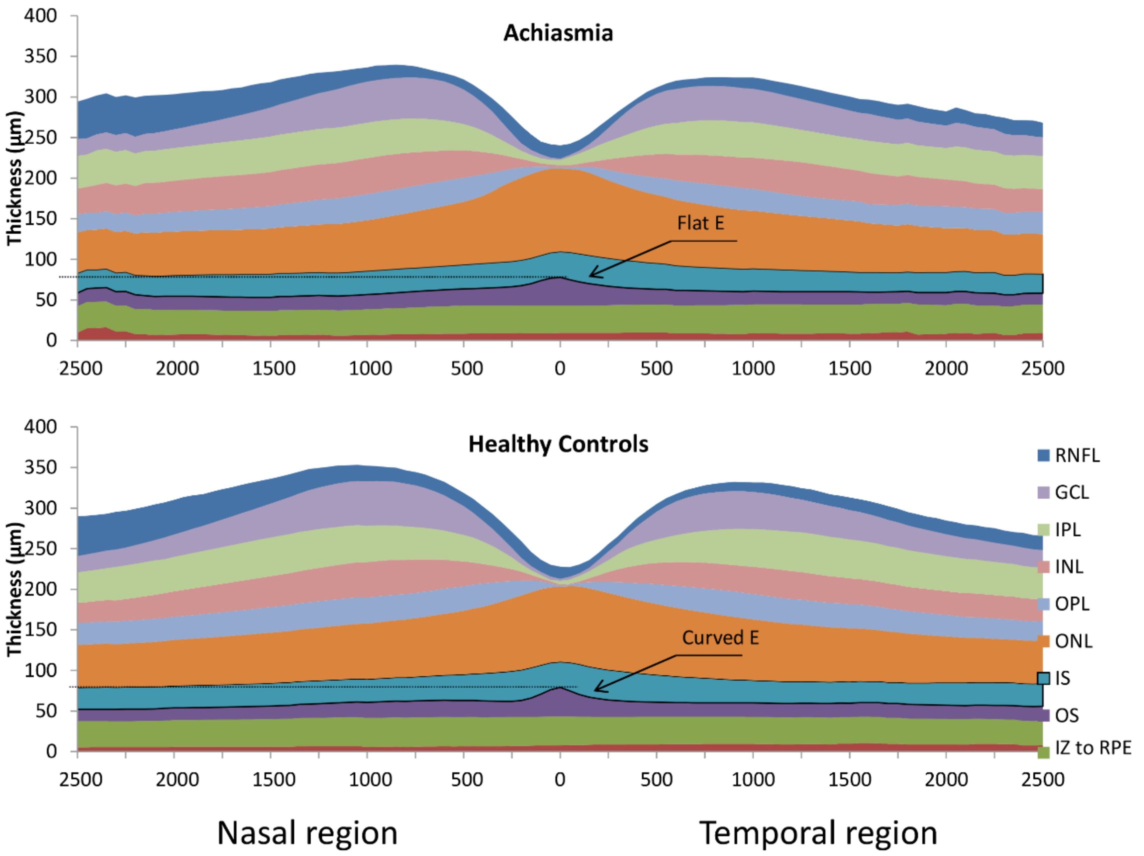

Supplementary figure 2: Cross sectional schematic diagram of mean parameters of individual retinal layers in patients with achiasmia and controls.

RNFL = nerve fibre layer; GCL = ganglion cell layer; IPL = inner plexiform layer; INL = inner nuclear layer; OPL = outer plexiform layer; ONL = outer nuclear layer; IS = inner segment; OS = outer segment; IZ = interdigitation zone; RPE = retinal pigment epithelium. Horizontal dotted line shows the peak of the ellipsoid line curve (E).

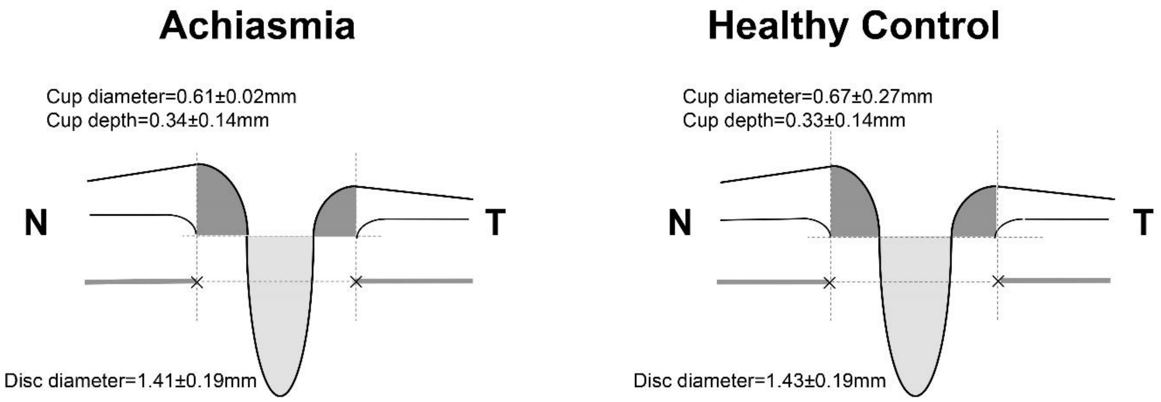

Supplementary figure 3: Cross-sectional schematic diagrams representing mean values of optic nerve head parameters of patients with achiasmia without ONH and healthy controls.

Upper horizontal dotted lines represent horizontal offset (150µm) used to determine cup diameters and the lower horizontal dotted lines indicate disc horizontal diameters. The vertical dotted lines show margins of rim areas. T = temporal and N = nasal.

No statistical significance was found.
